# Recurrent unintended pregnancies among young unmarried women serving in the Israeli military

**DOI:** 10.1186/s13584-018-0239-7

**Published:** 2018-12-31

**Authors:** Misgav Rottenstreich, Hen Y. Sela, Limor Loitner, Noam Smorgick, Zvi Vaknin

**Affiliations:** 1 Medical Corps, Israel Defense Forces, Jerusalem, Israel; 2 Department of Obstetrics and Gynecology, Shaare Zedek Medical Center, affiliated with the Hebrew University School of Medicine, Jerusalem, Israel; 3 Department of Obstetrics and Gynecology, Assaf Harofeh Medical Center, affiliated to Sackler School of Medicine, Tel Aviv University, Zerifin 70300, Beer Yakov, Israel

## Abstract

**Background:**

Unintended pregnancy is a major public health problem with known risk factors, however, little is known about the prevalence of variables associated with recurrent unintended pregnancy (RUP) among young, unmarried women.

**Methods:**

A retrospective cohort study of unmarried women aged 18–21 serving in the Israeli military between 2013 and 2015. Multivariable logistic regression analysis was used to examine associations between RUP and women’s education, IQ, immigration status, country of origin, socioeconomic status and history of psychiatric illness**.**

**Results:**

Of 129,638 women drafted by the Israeli military during the study period, 1720 women with unintended pregnancies had a follow up period of at least a year. Three hundred and eighty-nine of them had RUP (22.6%). Multivariable models comparing women with no unintended pregnancies and women with RUP revealed that RUP was more common among (adjusted relative risk; 95% confidence interval) women who had not graduated from high school (6.9; 4.99–9.55), who had low (90–99) IQ scores (3.9; 2.88–5.39) those reporting Africa as the country of origin (2.5; 1.37–4.59) and those from a lower socioeconomic neighborhood (1.6; 1.18–2.05). Multivariate regression modeling comparing women with single unintended pregnancies and women with RUPs showed that recurrent unintended pregnancy was more common among women who had not graduated from high school (3.2; 2.04–4.84) and those who had a low (90–99) IQ score (1.9; 1.32–2.61).

**Conclusion:**

Rate of RUP is high among women serving in the Israeli military. These women have unique epidemiological characteristics. This may serve in identifying populations at high risk and thus may enable policy maker to offer at least to this population Long-Acting Reversible Contraception (LARC) methods. We encourage policy makers to consider the provision of LARC methods to all servicewomen who had an unintended pregnancy.

## Background

Unintended pregnancies are a serious public health burden worldwide [1], and despite extensive efforts on reducing unintended pregnancies, nearly half of all pregnancies in the United States (US) in recent years were unintended [2].

The correlation between unmarried status, low income, ethnic minorities and lower education to unintended pregnancies has been examined in many studies, [2–4], however, less is known about risk factors for recurrent unintended pregnancies (RUP).

In our previous publication we performed a retrospective cohort study in order to identify the prevalence of and variables associated with unintended pregnancy among women serving in the Israeli military [5]. Women with single and recurrent unintended pregnancy were analyzed together and were compared to women without unintended pregnancy. We found the rate of unintended pregnancies to be lower than reported in most of the world. Failure to complete high-school and first-generation immigration were the most significant risk factors for having an unintended pregnancy during military service.

The Israeli army has mandatory educational programs that are held twice a year for all soldiers. These sessions focus on sexually transmitted diseases, contraception, unintended pregnancies prevention and treatment. All women soldiers have free and accessible visits upon demand to a gynecological clinic, where contraceptive counselling is held, and upon request oral contraceptive are prescribed. These prescriptions may be renewed with a general military physician and free of charge when purchased from military pharmacies. However, other methods of contraception such as subdermal implants and intrauterine contraceptives device are less easily accessible and are not free of charge. Even history of an unintended pregnancy is not an indication for free Long-Acting Reversible Contraception (LARC) and these women receive no special intervention regarding the prevention of the next pregnancy.

Few studies focus on the incidence and characteristics of RUP. According to the literature, the reported percentage of RUP among overall unintended pregnancies was estimated to be between 11 to 40% [6–8]. History of a previous unintended pregnancy is a risk factor for a subsequent unintended pregnancy primarily due to low rate of contraceptive use [6, 7].

The aim of this study was to estimate the recent rate of RUP among young, unmarried women enlisted in the Israeli military and to identify variables associated with the increased risk of RUP within in this population. These findings can be used to guide future efforts for secondary prevention among enlisted women with unintended pregnancies.

## Methods

A retrospective cohort study of all enlisted women in the Israeli Defense Force (IDF) drafted between January 2013 and December 2015 was performed by linking data from the Military Pregnancy Center (MPC) to data from the IDF Draft Board. One or more years of follow-up data were available for all cohort members included in the study. The study was approved by the Israel Defense Forces’ (IDF) Institutional Review Board.

The methodology of the cohort has been previously described in detail [5]. Unintended pregnancies and RUPs were identified in the MPC registry. History obtained at the time of creation of the MPC included the direct questions: “Is this pregnancy intended?” and “Is this your first pregnancy?”, thus allowing each pregnancy to be categorized as intended or unintended and primary or recurrent. Women who had more than two unintended pregnancies were also included in the analysis, however, because of their small number we didn’t analyzed them separately. Pregnancy recurrence was automatically assessed when a prior record was found in the MPC. Excluded from this analysis were women with intended pregnancies (*n* = 5), women whose pregnancies were a result of sexual assault (*n* = 6) and women without at least 1 year follow up (*n* = 645).

Variables were extracted and categorized from the Draft Board database and were evaluated for association with RUPs. These included: country of origin, immigration status, education level, Intelligence Quotient (IQ) score, neighborhood socioeconomic status and history of mental illness. The description of these variables is detailed in our previous publication [5], and is outlined briefly in this article.

Country of origin (classified by the father’s country of birth or if the father was born in Israel, by the paternal grandfather’s country of birth) was categorized into seven geographical areas: (1) Asia, (2) North Africa, (3) Sub-Saharan Africa (excluding South Africa), (4) Western (Europe, USA, Canada, Australia and South Africa), (5) former Soviet Union countries, (6) Israel, and (7) other (i.e. South American). Immigration status was categorized as first generation, second generation or longer-term resident. Neighborhood socioeconomic status (SES) was based on an index developed by the Israel Central Bureau of Statistics and validated previously. Briefly: Israel is divided into homogeneous “geographical units” and each area receives an ordinal ranking from 1 to 10 (low to high SES, respectively) [9]. History of mental illness was determined using Draft Board data, which was coded according to International Classification of Diseases (ICD-10) criteria [10] for personality disorders, affective disorders, anxiety disorders, etc.

Rate of RUP was calculated. We compared women reporting a recurrent unintended pregnancy to contemporary women soldiers who did not experience a pregnancy and to women reporting single unintended pregnancy using unpaired t tests to compare means and *χ*2 or the Fisher’s exact tests, as indicated, for categorical variables. We then used a multivariable generalized linear model with a binary logistic dependent variable to examine the relationships between risk of unintended pregnancy and women’s level of education, IQ, immigration status, country of origin, Body Mass Index (BMI), neighborhood SES and history of psychiatric illness. The results for these models were summarized by relative risk (reported as RR and 95% confidence intervals), with *p* < 0.05 considered statistically significant. All statistical analyses used SPSS 23.0.

## Results

**Fig. 1:**
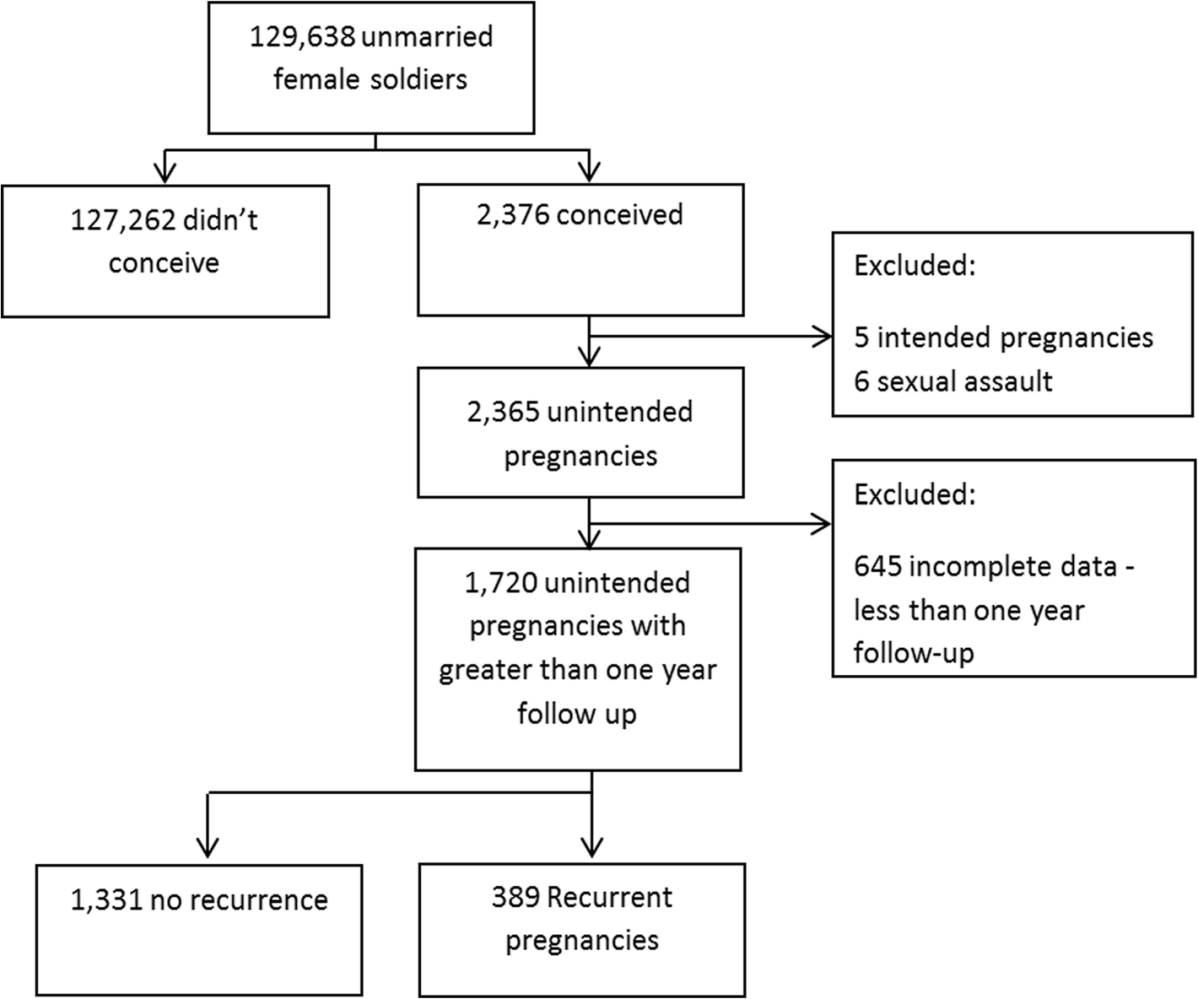
Schematic Study Flowchart

Of 129,638 young, unmarried women drafted by the Israeli military between 2013 and 2015, 2376 conceived, of these 645 women had less than 1 year follow-up and were excluded from the study cohort, majority (545) because of discharge from military service due their decision to continue the pregnancy to term. Therefore, we included 1720 women in the final analysis, 1331 women had only one unintended pregnancy and 389 reported recurrent unintended pregnancies. RUPs included in our study comprised 22.6% of the unintended pregnancies in the IDF during the study period with at least a year of follow and 0.3% of the overall female military population (Fig. 1).

Comparing women with RUP to women without unintended pregnancies using bivariate analyses revealed that (Relative Risk; 95% confidence interval) women with RUP were more likely to fail to graduate high school prior to joining the IDF (10.96; 8.59–13.98), had lower IQ scores (6.30; 4.93–8.07), were a first or second-generation immigrant (2.11; 1.62–2.75 and 1.54; 1.23–1.94, respectively), originate from Sub-Saharan Africa or the Former Soviet Union (9.36; 6.22–14.08 and 2.03; 1.37–3.02, respectively) and were more likely to come from neighborhoods with lower SES (1.85; 1.48–2.33). A history of psychiatric illness was not associated with an increased risk for recurrent unintended pregnancy. After controlling for other covariates, the variables most strongly associated with risk of recurrent unintended pregnancies (Table 1) were (adjusted Relative Risk; 95% confidence interval) failure to graduate from high school (6.9; 4.99–9.55), low (90–99) IQ score (3.9; 2.88–5.39), African country of origin (2.5; 1.37–4.59) and resided on neighborhoods with lower SES (1.56; 1.18–2.05).

**Table 1:** Variables associated with recurrent unintended pregnancy among women serving in the IDF, 2013–2015

	No pregnancy	Recurrent unintended pregnancy	Unadjusted Relative risk	Adjusted^a^ Relative Risk
	*n* = 127, 262	*n* = 389	(95% CI)	(95% CI)
Body Mass Index				
< 18.5	13.0%	17.1%	1.43 (1.08–1.90)	1.27 (0.90–1.790)
18.5–24.9	64.8%	59.6%	Reference	Reference
25–29.9	16.4%	18.4%	1.22 (0.93–1.60)	0.97 (0.69–1.37)
> 30	5.9%	4.9%	0.90 (0.56–1.45)	0.66 (0.37–1.16)
Education				
Less than high school	2.5%	21.9%	10.96 (8.59–13.98)	6.90 (4.99–9.55)
High school graduate	96.6%	77.1%	Reference	Reference
Higher education	0.9%	1.0%	1.36 (0.51–3.66)	N/A
IQ score				
Low (90–99)	33.8%	78.4%	6.30 (4.93–8.07)	3.94 (2.88–5.39)
Median (100–117.9)	56.4%	20.8%	Reference	Reference
High (118+)	9.8%	0.8%	0.22 (0.07–0.69)	0.12 (0.02–0.86)
Immigration status				
1st generation	9.0%	17.2%	2.11 (1.62–2.75)	1.50 (0.84–2.67)
2nd generation	34.5%	44.8%	1.54 (1.23–1.94)	1.18 (0.86–1.62)
Longer term resident			Reference	Reference
Country of origin				
Israel	16.8%	9.7%	Reference	Reference
Western	20.8%	9.4%	0.79 (0.49–1.25)	0.81 (0.47–1.37)
Sub-Saharan Africa	3.2%	17.5%	9.36 (6.29–14.08)	2.51 (1.37–4.59)
Asia	19.4%	13.7%	1.23 (0.80–1.88)	0.84 (0.53–1.35)
Former Soviet Union	18.3%	21.6%	2.03 (1.37–3.02)	0.91 (0.52–1.59)
North-Africa	21.2%	28.0%	1.57 (0.76–3.35)	1.28 (0.84–1.94)
Other	0.3%	0.0%	N/A	N/A
Neighborhood Socioeconomic Status				
Low (1–4)	20.1%	36.3%	1.85 (1.48–2.33)	1.56 (1.18–2.05)
Median (5–7)	55.4%	54.1%	Reference	Reference
High (8–10)	24.4%	9.6%	0.40 (0.28–0.58)	0.60 (0.38–0.93)
History of psychiatric illness	1.3%	1.3%	0.97 (0.40–2.35)	1.11 (0.41–3.02)

*N/A* not available - numbers are too small for calculation

r^a^Adjusted for all variables shown in table

Comparison of women with RUP with those with single unintended pregnancy using bivariate analyses revealed that women with recurrent unintended pregnancies (Relative Risk; 95% confidence interval) were less likely to have graduated from high school prior to joining the IDF (3.40; 2.48–4.66), had lower IQ scores (2.28; 1.75–3.00), had lower BMI (1.49; 1.08–2.07), had African country of origin (1.81; 1.13–2.89) and were more likely to come from neighborhoods with lower SES (1.34; 1.03–1.74). After controlling for other covariates, the variables most strongly associated with risk of recurrent unintended pregnancies (Table 2) were (adjusted Relative Risk; 95% confidence interval) failure to graduate from high school (3.16; 2.04–4.84) and low IQ score (1.86; 1.32–2.61).

**Table 2:** Relative risks for single versus recurrent unintended pregnancy among women serving in the IDF, 2013–2015

	Single unintended pregnancy	Recurrent unintended pregnancy	Unadjusted RR (95% CI)	Adjusted^a^ RR (95% CI)
	*n* = 1331 (77.4%)	*n* = 389 (22.6%)		
Body Mass Index				
< 18.5	12.4%	17.1%	1.49 (1.08–2.07)	1.41 (0.93–2.16)
18.5–24.9	64.5%	59.6%	Reference	Reference
25–29.9	16.9%	18.4%	1.18 (0.87–1.61)	0.93 (0.62–1.39)
> 30	6.1%	4.9%	0.86 (0.51–1.47)	0.81 (0.42–1.58)
Education				
Less than high school	7.7%	21.9%	3.40 (2.48–4.66)	3.15 (2.04–4.84)
High school graduate	92.0%	77.1%	Reference	Reference
Higher education	0.4%	1.0%	3.26 (0.87–12.23)	N/A
IQ score				
Low (90–99)	60.7%	78.4%	2.29 (1.75–3.00)	1.86 (1.32–2.62)
Median (100–117.9)	36.8%	20.8%	Reference	Reference
High (118+)	2.5%	0.8%	0.55 (0.17–1.84)	0.27 (0.04–2.17)
Immigration status				
1st generation	16.8%	17.2%	1.03(0.76–1.39)	0.69 (0.10–0.27)
2nd generation	38.6%	44.8%	1.29 (0.997–1.67)	1.30 (0.90–1.88)
Longer term resident			Reference	Reference
Country of origin				
Israel	10.9%	9.7%	Reference	Reference
Western	14.1%	9.4%	0.75 (0.45–1.25)	0.76 (0.42–1.39)
Sub-Saharan Africa	10.9%	17.5%	1.81 (1.13–2.89)	1.44 (0.69–3.00)
Asia	16.7%	13.7%	0.92 (0.57–1.48)	0.74 (0.43–1.27)
Former Soviet Union	20.1%	21.6%	1.20 (0.77–1.87)	0.83 (0.43–1.61)
North-Africa	27.1%	28.0%	1.16 (0.76–1.78)	0.86 (0.53–1.41)
Other	0.2%	0.0%	N/A	N/A
Neighborhood Socioeconomic Status				
Low (1–4)	28.9%	36.3%	1.34 (1.03–1.74)	1.13 (0.82–1.57)
Median (5–7)	57.6%	54.1%	Reference	Reference
High (8–10)	13.5%	9.6%	0.76 (0.51–1.14)	0.68 (0.41–1.13)
History of psychiatric illness	1.8%	1.3%	0.71 (0.27–1.87)	0.64 (0.20–2.06)

*N/A* not available - numbers are too small for calculation

^a^Adjusted for all variables shown in table

## Discussion

This study found that the rate of RUP among women with unintended pregnancy, with at least 1 year of follow up, serving in the Israeli military was 22.6% during the study period. Our findings are consistent with other previous studies. The rate of RUPs delivered in a university hospital in Ireland and followed for 3-years was 11.4% (124/1087) [6], among US women aged 14–35 followed over 2-years in the Project PROTECT was 27% (33/122) [7] and among Japanese women aged 35–49 was 40.1% (78/195) [8]. These differences may be influenced by age, follow up period, and other cultural factors related to the population studied.

This study is among the first to examine the factors associated with RUP. Previously it was reported that RUP is mainly due to low rates of contraceptive use [6], We found that in our study population, RUP was most common among women who did not graduate from high school prior to joining the IDF, who had a low IQ-score, resided in a neighborhood with a low SES and country of origin was Sub-Saharan Africa. However, we can only speculate about the explanation between these risk factors and the recurrence of unintended pregnancies, such as knowledge regarding contraception use and compliance.

Failure to graduate from high school and a low IQ score were also found to be independently associated with RUP when compared to women with only one unintended pregnancy during the study period. These findings further emphasize the role of education as a proactive approach of secondary prevention in this unique sub-group of soldiers.

The relationship between unintended pregnancy and maternal BMI has been studied previously, however, the findings were inconsistent [11, 12]. Increased risks for unintended pregnancies, among both underweight and obese women were related to high rates of non-hormonal contraceptive use or hormonal contraceptive failure. In our study, we found higher risks of unintended pregnancy in underweight women (BMI < 18.5 m/kg2) compared with those of normal BMI, however, this association weakened considerably after adjustment for other variables.

The findings of this study may serve in identifying populations at high risk and thus may enable policy maker to consider based on a cost benefit analysis few options: 1. Continue current practice and having to cope with 22% of RUP (which translates to roughly 120 annual RUP) 2. Offer LARC to all women with one previous unintended pregnancy (this translates to roughly 600 women annually) 3. Offer at least high risk population use of LARC methods (this translates to 70% of women with one unintended pregnancy – roughly 400 women annually). We encourage policy makers to consider these options.

Our study has strengths and limitations. The current study relied on the data available for analysis; it is possible that other factors that were not explored may have a much greater impact on RUPs in this population, e.g. contraceptive use, believes and knowledge about contraceptive use, sexual activity and religious status. Another limitation is the relatively short follow-up period and it is possible that more women conceived after the 1-year follow-up or after completion of the 2 year mandatory service. Strengths of this study included the large size of the cohort, the reliable prospective nature of data entry into the military databases, and the focus on unintended pregnancy rather than unintended births. The study is the first to describe the unique characteristics of women with RUPs.

## Conclusions

In conclusion, this study shows that almost quarter of the unmarried young women serving in the IDF who experiences an unintended pregnancy, had a subsequent unintended pregnancy. Women with RUP have unique demographic and social characteristics. Secondary prevention, to further reduce the rates of RUPs should focus on women who have not graduated from high school prior to joining the IDF and women with a low IQ score. The findings of this study, may serve in identifying populations at high risk and thus may enable policy maker to offer at least to this population LARC methods. We encourage policy makers to consider the provision of LARC methods to all servicewomen who had an unintended pregnancy.

## Abbreviations

BMI: Body mass index
ICD: International classification of diseases
IDF: Israeli defense force
IQ: Intelligence quotient
LARC: Long-acting reversible contraception
MPC: Military pregnancy center
RR: Relative risk
RUP: recurrent unintended pregnancy
SES: Socioeconomic status
US: United States
